# Influence of the second stage of labor on maternal and neonatal outcomes in vaginal births after caesarean section: a multicenter study in Germany

**DOI:** 10.1186/s12884-021-03817-2

**Published:** 2021-05-04

**Authors:** G. Gitas, L. Proppe, A. K. Ertan, S. Baum, A. Rody, M. Kocaer, K. Dinas, L. Allahqoli, A. S. Laganà, A. Sotiriadis, S. Sommer, I. Alkatout

**Affiliations:** 1 Department of Obstetrics and Gynecology, University Hospital Schleswig Holstein, Campus Luebeck, Ratzeburger Allee 160, Haus A, 23538 Luebeck, Germany; 2 Department of Obstetrics and Gynecology, Leverkusen Municipal Hospital, Leverkusen, 51375 Germany; 3 Second Department of Obstetrics and Gynecology, Medical School, Aristotle University of Thessaloniki, Konstaninoupoleos 49, Thessaloniki, 546 42 Greece; 4 School of Public Health, Iran University of Medical Sciences (IUMS), Tehran, 14535 Iran; 5 Department of Obstetrics and Gynecology, Filippo Del Ponte Hospital, University of Insubria, Varese, Italy; 6 Department of Obstetrics and Gynecology, University Hospital Schleswig Holstein, Campus Kiel, Kiel, Germany

**Keywords:** TOLAC, VBAC, Prolonged second stage, Maternal outcome, Neonatal outcome

## Abstract

**Background:**

The American College of Obstetricians and Gynecologists (ACOG) introduced a new standard of care in 2014, extending the duration of the second stage of labor in order to reduce caesarean delivery (CD) rates and its severe complications. The aim of the present study is to evaluate success rates of trial of labor after caesarean section (TOLAC), as well as maternal and neonatal outcomes after the establishment of the recent guidelines.

**Methods:**

A retrospective study was performed at two large departments in Germany from January 2008 to January 2018. Patients undergoing TOLAC were divided into two groups. Group I (958 patients) was constituted before the establishment of the current guidelines, and Group II (588 patients) after the establishment of the guidelines. A subgroup analysis was performed to compare neonatal outcomes after successful TOLAC and operative vaginal delivery with those after failed TOLAC and secondary CD.

**Results:**

The success rate of vaginal births after cesarean section (VBAC) fell from 66.4% in Group I to 55.8% in Group II (*p* < 0.001). The median duration of the second stage of labor was statistically significantly longer in Group II than in Group I (79.3 ± 61.9 vs. 69.3 ± 58.2 min) for patients without previous vaginal birth. The incidence of operative vaginal delivery decreased from Group I to Group II (9.6% vs. 6.8%). The incidence of third- and fourth-degree perineal lacerations, blood loss and emergency CD were similar in the two groups. Concerning the neonatal outcome, our groups did not differ significantly in regard of rates of umbilical artery cord pH < 7.1 (*p* = 0.108), the 5-min Apgar scores below 7 (*p* = 0.224) and intubation (*p* = 0.547). However, the transfer rates to the neonatal care unit were significantly higher in Group II than in Group I (*p* < 0.001). Neonatal outcomes did not differ significantly in the subgroup analysis.

**Conclusion:**

Extending the second stage of labor does not necessarily result in more vaginal births after TOLAC. Maternal and neonatal outcomes were similar in both groups. Further studies will be needed to evaluate the role of operative vaginal delivery and the duration of the second stage of labor in TOLAC.

## Background

There has been a notable increase in the number of caesarean deliveries (CD) over the last decade throughout the world. In the United States, CD rates rose from 5% of all deliveries in 1970 to 31.9% in 2016 [1]. In some countries, a CD is considered necessary or is offered to women who have had previous caesarean sections. This has contributed to the overall increase in CD rates [2]. Thus, the trial of labor after caesarean section (TOLAC) is an essential strategy to reduce CD rates. Vaginal birth after caesarean section (VBAC) is achieved in 65 to 83.3% of cases [3, 4]. Although VBAC is a safe medical procedure, VBAC rates have declined throughout the world in the last few years. Conversely, the rate of elective repeat caesarean section (ERCS) is on the rise [5]. In the United States, the overall rate of VBAC fell from 24% in 1996 to 8% in 2010, which is a matter of public and professional concern [6]. One reason for this phenomenon may be ambiguous evidence of the risks of VBAC, which causes fear and anxiety in patients [7].

In general, CD is associated with more severe maternal complications [8] compared to vaginal deliveries. The benefits of vaginal birth, such as rapid maternal recovery, fewer maternal complications in future pregnancies, and a potentially lower risk of childhood diseases (such as allergies, asthma, or obesity) should be taken into account [9]. A number of studies published in recent times have addressed the outcome of TOLAC and yielded variable results. However, VBAC is overall relatively safe for mother and child compared to ERCS [10]. Successful VBACs have been associated with lower overall morbidity rates [11] compared to ERCS. However, a failed VBAC is associated with a higher risk of perinatal and maternal complications compared to ERCS [12].

Based on guidelines published by the ACOG (American College of Obstetricians and Gynecologists) and SMFM (Society for Maternal Fetal Medicine) in 2014 [13], many hospitals throughout the world have introduced a new standard of care concerning the duration of the second stage of labor in order to reduce CD rates. According to these recommendations, the second stage of labor may take an indefinite period of time, provided the delivery progresses well, and maternal and fetal wellbeing are ensured. This statement has been endorsed by other recommendations [14, 15] and studies [16]. In contrast, the previous guidelines [17, 18] recommended a maximum duration of approximately 1 or 2 h in multiparous women for the second stage of labor, depending on whether or not a regional anesthesia was performed. The correlation between the duration of the second stage of labor and adverse maternal and neonatal outcomes has been investigated in several studies [19, 20].

The purpose of the present study is to investigate the consequences of the most recent ACOG/SMFM guidelines with respect to success rates of TOLAC. We also analyzed maternal and fetal outcomes.

## Material and methods

A retrospective multicenter study was performed at two large departments of obstetrics and gynecology in Germany, including patients with high-risk pregnancies, from January 2008 to January 2018. The study was performed in compliance with the Helsinki declaration, and was approved by the medical ethics committee. The hospital information systems of the academic teaching hospitals of Klinikum Leverkusen and the University Hospital of Luebeck in Germany were used to identify eligible patients. Inclusion criteria were defined as singleton pregnancy, a history of only one previous caesarean delivery with a low transverse incision, a viable fetus in cephalic presentation, patients > 32 weeks of gestation [a vaginal delivery under this gestational age was not favored earlier at the above mentioned our institutions [21–23]], and the intention to deliver by the vaginal route.

**Fig. 1:**
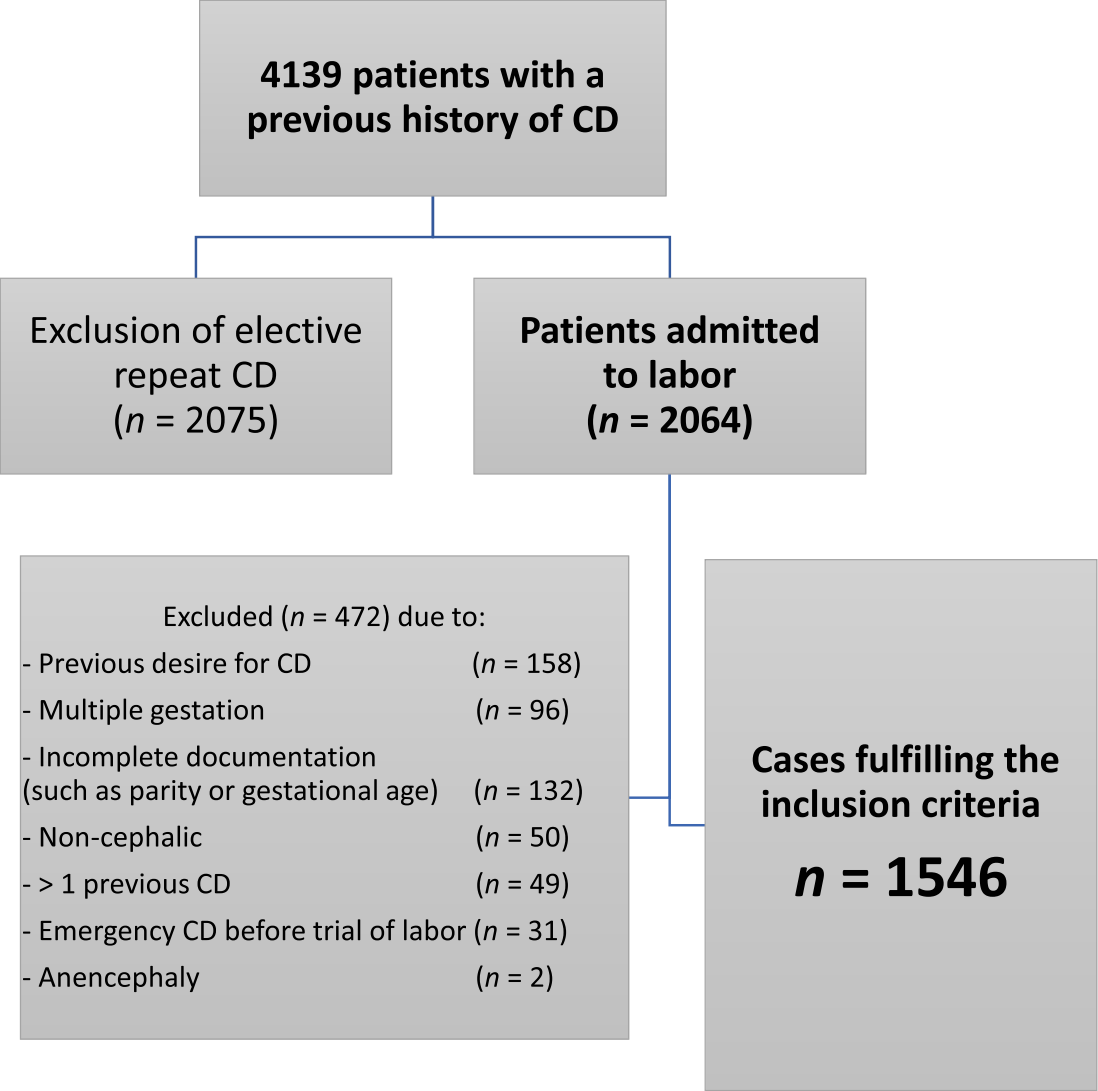
Flowchart through the recruitment phase of the study

A computer-based search yielded 4139 patients with only one previous caesarean section in their medical history. Approximately a half of them had undergone an elective repeat caesarean section. Further exclusion criteria were emergencies before labor, intrauterine growth restriction, fetal anomalies, and multiple gestation. Finally, 1546 patients (607 from Leverkusen and 939 from Luebeck) fulfilled the inclusion criteria (Fig. 1).

Parity, clinical parameters such as age, body mass index (BMI), gestational age, preterm birth, the occurrence of a vaginal birth or VBAC before TOLAC, gestational diabetes, and hypertensive pregnancy-related diseases were analyzed. Maternal surveillance data such as birth analgesia, labor induction, maternal outcomes (estimated blood loss, emergency CD, uterine rupture, third- and fourth-degree perineal tears, episiotomy, postoperative hysterectomies), and fetal outcomes (fetal weight, umbilical artery cord pH < 7.1 or < 7.0, 5-min Apgar scores below 6 or 7, intubation rate, and transfer to neonatal care unit) were analyzed. Oxytocin or prostaglandin were used for the induction of labour. The exact duration of the second stage of labor was registered. Both departments adopted the guidelines published by the ACOG in 2014 concerning the duration of second stage of labor and were universally carried out, Furthermore, the same standards of care were maintained at both institutions. A subgroup analysis was performed to compare neonatal outcomes after VBAC with vaginal operative delivery and failed TOLAC with secondary CD.

The patients were divided into two groups in order to compare VBAC, maternal and fetal outcomes after TOLAC in relation to the duration of the second stage of labor. Group I (958 patients) included deliveries from January 2008 to March 2014 at the University Hospital of Luebeck, and from January 2008 to March 2014 at the academic teaching hospitals of Klinikum Leverkusen (time period I). Deliveries from April 2014 to January 2018 (time period II), constituted Group II (588 patients). The second stage of labor was defined as the period of time from complete dilatation of the cervix to the delivery of the infant. During time period I, a second-stage arrest was established when the second stage persisted for 2 or 3 h, required regional anesthesia in nulliparous women, and a caesarean section had to be considered. In multiparous women, a second-stage arrest was established after 1 or 2 h, coupled with a need for regional anesthesia [17, 18]. In Group II, based on new standards published in 2012 [15] and 2014 [13], no absolute maximum length of time was defined for the second stage of labor; at least one additional hour elapsed before a second-stage arrest was established. A subgroup analysis was performed to compare neonatal outcomes after VBAC with operative vaginal delivery and failed TOLAC with secondary CD.

Statistical analysis was performed using the SPSS software. Continuous and categorical variables were shown as numbers of patients and percentages. Depending on the scaling and distribution of the variables considered, either a chi-square-test or a Fisher’s exact test was performed. *P*-values less than or equal to 0.05 were considered statistically significant.

## Results

Baseline characteristics and maternal surveillance were similar in both groups (958 women in Group I and 588 women in Group II) (Table 1). The median duration of the second stage of labor was longer in Group II (65.4 ± 60.7 min) than in Group I (57.4 ± 56.3 min), but the difference was not statistically significant (*p* = 0.098). However, the median duration of the second stage of labor was significantly longer in patients without previous vaginal delivery (79.3 ± 61.9 min in Group II vs. 69.3 ± 58.2 min in Group I) (*p* = 0.045).

**Table 1:** Patient characteristics

	Group I (*n* = 958)	Group II (*n* = 588)	Total (*n* = 1564)	***p***
Age (years)	32.5 ± 5.1	32.5 ± 4.9	32.5 ± 5.0	**0.702**
Parity	2.4 ± 0.8	2.3 ± 0.8	2.4 ± 0.80	**0.055**
Gestational age (weeks)	39.0 ± 2.1	39.0 ± 2.1	39.1 ± 2.1	**0.182**
BMI (kg/m^2^)	25.4 ± 5.6	26.0 ± 6.2	25.7 ± 5.8	**0.197**
Previous vaginal birth	211 (28.7%)	136 (23.6%)	347 (26.4%)	**0.036**
Prostaglandin used	264 (27.6%)	213 (36.2%)	477 (30.9%)	**< 0.001**
Oxytocin used	410 (42.9%)	160 (27.2%)	570 (36.9%)	**< 0.001**
Oxytocin and prostaglandin used	117 (12.2%)	61 (10.4%)	178 (11.5%)	**0.271**
Gestational diabetes	110 (11.5%)	61 (10.4%)	171 (11.1%)	**0.503**
Hypertensive, pregnancy-related disease	51 (5.3%)	34 (5.8%)	85 (5.5%)	**0.699**
Birthweight of previous infant delivered by CD (g)	3049 ± 811	3059 ± 816	3052 ± 811	**0.649**
Neonatal weight (g)	3351 ± 587	3332 ± 586	3344 ± 586	**0.757**
Preterm birth (≤ 36 + 6 weeks of gestation)	114 (11.9%)	72 (12.2%)	186 (12.0%)	**0.840**
Median duration of the second stage of labor (minutes) (*n* = 789)	57.4 ± 56.3	65.4 ± 60.7	61.0 ± 58.5	**0.098**
Median duration of the second stage of labor (minutes) for patients with previous vaginal birth (*n* = 227)	28.3 ± 38.3	31.82 ± 42.3	29.91 ± 40.1	**0.910**
Median duration of the second stage of labor (minutes) for patients without previous vaginal birth (*n* = 560)	69.3 ± 58.2	79.3 ± 61.9	73.8 ± 60.0	**0.045**

As shown in Table 2, the rate of successful TOLAC fell from 66.4% (636/958 patients) in Group I to 55.6% (327/588) in Group II (*p* < 0.001), while CD rates increased from 33.7 to 43.7% (*p* < 0.001). Vacuum-assisted deliveries accounted for 13.7% of vaginal, and 8.5% of total deliveries. The incidence of operative vaginal deliveries fell from Group I to Group II (92 to 40 patients in total; 9.2% vs. 6.8%; *p =* 0.03).

**Table 2:** Maternal and neonatal outcome

	Group I (*n* = 958)	Group II (*n* = 588)	Total (*n* = 1564)	*p*
Successful TOLAC = VBAC	636 (66.4%)	327 (55.6%)	963 (62.3%)	**< 0.001**
Vaginal operative delivery	92 (9.6%)	40 (6.8%)	132 (8.5%)	**0.003**
Secondary cesarean section	323 (33.7%)	257 (43.7%)	580 (37.5%)	**< 0.001**
Episiotomy	215 (22.4%)	79 (13.4%)	294 (19.0%)	**< 0.001**
Third- or fourth degree of perineal laceration	9 (0.9%)	7 (1.2%)	16 (1.0%)	**0.636**
Blood loss (ml)	333 ± 388	331 ± 204	332 ± 319	**0.095**
Emergency CD	10 (1.9%)	11 (2.6%)	21 (2.2%)	**0.449**
Uterine rupture	10 (1.0%)	10 (1.7%)	20 (1.3%)	**0.267**
Apgar score at 1 min (mean)	8.46 ± 1.28	8.50 ± 1.42	8.48 ± 1.33	**0.017**
Apgar score at 5 min (mean)	9.50 ± 0.97	9.55 ± 1.05	9.52 ± 1.00	**0.011**
Apgar score at 10 min (mean)	9.78 ± 0.77	9.73 ± 0.95	9.75 ± 0.85	**0.854**
Umbilical cord pH	7.30 ± 0.09	7.28 ± 0.09	7.29 ± 0.09	**< 0.001**
Base excess	−3.05 ± 3.32	−3.04 ± 3.51	−3.04 ± 3.41	**0.714**
Transfer to neonatal care unit	19 (4.4%)	20 (11.7%)	39 ± 6.5%	**< 0.001**
Intubation	7 (1.2%)	8 (1.7%)	15 ± 1.4%	**0.547**
5-min Apgar score below 6	8 (0.8%)	7 (1.2%)	15 (1.0%)	**0.489**
5-min Apgar score below 7	12 (1.3%)	12 (2.0%)	24 (1.6%)	**0.224**
pH < 7.1	10 (1.0%)	12 (2.0%)	22 (1.4%)	**0.108**
pH < 7.0	3 (0.3%)	5 (0.9%)	8 (0.5%)	**0.166**

With regard to maternal outcome (Table 2), no differences were registered in respect of emergency CD’s, uterine rupture, third- and fourth-degree perineal and severe vaginal lacerations, and blood loss by extending the second stage of labor. Two postpartum hysterectomies were performed (one in each group).

With regard to neonatal outcomes, the umbilical artery cord pH < 7.1 and the 5-min Apgar scores below 6 or 7 did not have a statistically significant difference between the two groups. Furthermore, they presented similar Apgar scores at 10 min (*p* = 0.854) and intubation rates (*p* = 0.547). On the other hand, transfer rates to the neonatal care unit were significantly higher in Group II than in Group I (*p* < 0.001) (Table 2).

A subgroup analysis of neonatal outcomes after vaginal operative delivery compared with secondary CD is shown in Table 3. The 5-min Apgar scores below 6 or 7 did not differ statistical significantly between the two groups. Moreover, intubation rates (*p* = 0.705), rates of transfer to the neonatal care unit (*p* = 0.371), and the frequency of an umbilical cord pH < 7.1 (*p* = 0.731) were similar in both groups.

**Table 3:** Neonatal outcome of vaginal operative delivery (VOD) in comparison with secondary caesarean section

	VOD (*n* = 135)	Secondary CD (*n* = 577)	Total (*n* = 712)	***p***
Apgar at 1 min (mean)	7.87 ± 1.59	8.13 ± 1.68	8.08 ± 1.66	**0.002**
Apgar at 5 min (mean)	9.27 ± 1.01	9.24 ± 1.20	9.25 ± 1.16	**0.904**
Apgar at 10 min (mean)	9.65 ± 0.67	9.60 ± 0.91	9.60 ± 0.88	**0.805**
pH	7.23 ± 0.10	7.31 ± 0.10	7.29 ± 0.10	**< 0.001**
pH < 7.1	3 (4.5%)	14 (3.8%)	17 (3.9%)	**0.731**
5-min Apgar score below 6	1 (0.8%)	10 (1.7%)	11 (1.5%)	**0.699**
5-min Apgar score below 7	3 (2.3%)	17 (2.9%)	20 (2.8%)	**1.000**
Base excess	−5.41 ± 3.51	−2.35 ± 3.89	−2.81 ± 3.99	**< 0.001**
Transfer to neonatal care unit	7 (10.6%)	15 (7.2%)	22 (8.0%)	**0.371**
Intubation	1 (1.2%)	12 (2.9%)	13 (2.6%)	**0.705**

*VOD* vaginal operative delivery, *CD* cesarean section

## Discussion

To our knowledge, this is the first study addressing the outcome of TOLAC with reference to the ACOG/SMFM recommendations [13]. A low CD rate is one of the prime objectives of obstetricians throughout the world [15]. Our data showed that adherence to the guidelines were associated with a significantly longer duration of the second stage of labor in patients without previous vaginal delivery, an unexpected fall in VBAC rates, and a rise in secondary CD rates after TOLAC. Moreover, the extension of the second stage of labor was associated with a significant fall in operative vaginal deliveries. Maternal and neonatal outcomes did not differ significantly between groups. Both groups were homogenous in regard of clinical parameters and outcome factors.

A few studies have addressed the effectiveness of the new labor guidelines to prevent primary CD in all women. The results were controversial. A study from Pennsylvania, USA, revealed a fall in CD rates from 26.9 to 18.8% in nulliparous patients after the new labor guidelines [24]. Zipori et al. included multiparous and nulliparous women in their analysis, and registered a decrease in CD rates from 23.3 to 15.7% [25]. By contrast, a study comprising 7845 patients [26] indicated that CD rates were not reduced after application of the new labor guidelines (15.8% vs. 17.7%). The management of the second stage of labor with the aim of preventing primary CD is apparently still a debated issue.

Our results were similar to the observational data obtained from the OptiBIRTH randomized trial [27]: 790 patients undergoing TOLAC from Ireland, Italy, and Germany were analyzed. Patients with a shorter duration of labor had more successful VBAC compared to those with a longer duration of labor. The first and second stage of labor in the OptiBIRTH trial was 4.68 vs. 7.83 h (*p* < 0.001), and 0.70 vs. 2.13 h (*p* < 0.001), respectively. Moreover, an earlier intrapartum intervention, such as amniotomy, was significantly associated with more VBAC (3.50 h vs. 6.08 h). The authors conclude that a shorter duration of labor favors VBAC, and the progression of labor may be assisted by stimulating endogenous uterine contractions. The effectiveness of stimulating endogenous uterine contractions could not be evaluated in the present study because of its retrospective design.

We observed a decrease in the total number of operative vaginal deliveries after the application of the new guidelines. This might have been due to the different approaches towards labor. With the aim of achieving delivery at the latest 2 h after full cervical dilatation, obstetricians may lead women actively through labor, whereas a longer second stage of labor might favor protraction and reduce uterine rupture rates. Additionally, a prolongation of the second stage may cause maternal exhaustion and favor secondary caesarean section rather than operative vaginal delivery. These circumstances may also cause fetal distress and thus contribute to a higher rate of secondary CD. Unfortunately, we could not address the success rate of vacuum birth in the two groups, which could help us to better interpretate our finding. It is not clear if the physicians performed failed or less vacuum births if the second stage was already long. However, according to our experience, a failed vacuum birth is a rare case. Further research is needed to enlighten this field.

The safety and effectiveness of operative vaginal delivery in relation to the outcome and success of TOLAC were analyzed by Krizman et al. in 1837 patients [28]. Success rates of vaginal operative delivery in TOLAC were high (forceps 90.4%; vacuum 92.6%), and neonatal morbidity rates were similar to those for repeat cesarean delivery. With regard to overall morbidity the authors conclude that, in order to avoid secondary CD, TOLAC should be offered to all women with no apparent contraindications for vaginal delivery, and operative vaginal delivery might be considered more often than considered previously in these patients. Our study revealed that, after the establishment of the new guidelines, the number of operative vaginal deliveries fell from 9.2 to 6.8% and the number of secondary CD increased. These data confirm the conclusions of the above-mentioned study. Additionally, according to our subgroup analysis, operative vaginal delivery has similar neonatal outcome with the secondary CD. Analytically, the incidence of postpartum acidosis (pH < 7.1) were similar in our subgroups (4.5% vs 3.8%) but as expected, significant higher as by our main groups (1.0% Group I vs. 2.0% Group II). Furthermore, parameters which may be associated to long-term neonatal outcome, such as 5-min Apgar scores below 6 or 7 or Apgar scores at 10 min, did not differ statistical significantly between the two groups. Thus, if we take in considerations the negative maternal effects of CD [29], operative vaginal delivery should be preferred, when possible.

In a retrospective investigation performed in 2019, Miller et al. [30] concluded that an operative intervention might be considered after a two-hour duration of the second stage of labor without epidural anesthesia, a three-hour duration with epidural anesthesia, and a one-hour duration or less in women with a previous vaginal delivery, for those undergoing TOLAC. These measures are in accordance with former guidelines. According to the new guidelines [13, 15], the duration of the second stage of labor with and without epidural anesthesia could be at least 4 and 3 h, respectively. Zhang et al. [22] used an interval-censored regression to analyze labor curves and suggested that the 95th percentiles of the second stage of labor in nulliparous women were 3.6 and 2.8 h with and with without epidural anesthesia. However, only low-risk women were included in the investigation. Labor curves for high-risk women, such as those undergoing TOLAC, have not been thoroughly examined yet. As Zheng et al. suggested, the ideal time for the second stage of labor would differ by populations and may be shorter for patients undergoing TOLAC [31].

After application of the new guidelines, we observed a slight but not statistically significant increase in third- and fourth-degree perineal lacerations from 0.9 to 1.2%. Risk factors for third- and fourth-degree perineal lacerations [32], such as the mother’s ethnicity and infants large for their gestational age, were comparable in both groups. Furthermore, as the frequency of vaginal operative deliveries decreased after the establishment of the new guidelines, a reduction in third- and fourth-degree perineal lacerations could be expected. In a study comprising 19,831 patients, Zipori et al. [25] registered an increase in third- and fourth-degree perineal lacerations from 1 to 1.3% after application of the new labor guidelines. Third- and fourth-degree perineal lacerations exert a significant impact on the patients’ quality of life in terms of higher rates of dyspareunia, wound breakdown and infection, urinary incontinence, and postpartum depression secondary to perineal pain [33, 34].

Similar to maternal morbidity, neonatal outcomes did not differ markedly between Groups I and II in the present study. The incidence of postpartum acidosis (pH < 7.1) or severe metabolic acidosis (pH < 7.0) were similar in both groups. Further essential parameters, such as the 5-min Apgar scores below 6 or 7 or Apgar scores at 10 min, did not differ statistical significantly between the two groups. In our study, the proportion of 5-min Apgar scores below 7 (1.3% in Group I and 2.0% in Group II) were higher than the medial rate reported in the literature (less than 1%) [35], which could be expected due to our high-risk collective. On the other hand, transfer rates to the neonatal care unit were significantly higher in Group II than in Group I (11.7% vs. 4.4%). Unfortunately, we could not find a reasonable explanation about this difference, which may be associated with the development of a more safe and accurate neonatal examination and surveillance in the last years, an issue which seems to be multifactorial [36]. Further research is needed in order to enlighten this field.

Sepsis and infection rates were not included in this analysis because of the small number of cases. Moreover, it was not possible to analyze the rate of preeclampsia because of a change in diagnostic criteria during the study period. Two recent studies [37, 38] investigating morbidity and CD prevention rates after application of the new labor guidelines showed no association between the duration of the second stage of labor and maternal or neonatal morbidity. However, some studies revealed increased rates of chorioamnionitis, sepsis, uterine atony, and third- or fourth-degree perineal lacerations after a prolonged second stage of labor [16, 39]. A prolonged second stage with the aim of reducing CD rates may be associated with higher maternal and neonatal morbidity rates. Thus, this strategy is still a controversially discussed issue.

To our knowledge, the current study is the first to analyze the outcome of patients undergoing TOLAC before and after the new recommendations of the ACOG/SMFM [13], especially with regard to the duration of the second stage of labor. Statistical bias was reduced by the fact that the present investigation was a multicenter study in a large population with homogenous groups. However, the limitations of the study are worthy of mention. The retrospective design called for a careful interpretation of the results. Factors such as physician’s or midwife’s experience and preferences were not assessed, and might have accounted for the results. Furthermore, the retrospective design of the present study provided a relatively large study population, but was limited by the documentation performed during the deliveries. Moreover, the size of the study didn’t not allow us to perform a multivariate regression analysis, which may negatively influence our results and increase the risk of statistical bias. On the other hand, after the establishment of the new guidelines, it will be difficult to perform a prospective study with sufficient numbers of patients.

## Conclusions

The present study provides new data about the outcome of TOLAC after the establishment of the ACOG/SMFM recommendations concerning the duration of the second stage of labor. Notably, the investigation revealed that a prolonged second stage of labor does not necessarily lead to more vaginal births after TOLAC. We also observed a reduction in vaginal operative deliveries. Further studies will have to evaluate the role of the second stage of labor on CD rates, especially in TOLAC. Several thousands of patients will be needed to obtain statistically robust data concerning severe maternal and neonatal complications. Moreover, prospective studies will be likely to provide valuable data on the care of women with VBAC. Women undergoing TOLAC are known to be subject to a higher risk of obstetric complications; normal labor curves have not been assessed for them. Based on the existing data, we conclude that women who have no contraindications for vaginal delivery should be encouraged to undergo TOLAC after a careful medical assessment. This should be accompanied by careful monitoring at well-equipped centers with facilities for immediate operative procedures.

## Data Availability

The datasets used and analyzed during the current study are available from the corresponding author on reasonable request.

## Abbreviations

ACOG: American College of Obstetricians and Gynecologists
CD: Caesarean delivery
ERCS: Elective repeat caesarean section
SMFM: Society for Maternal Fetal Medicine
TOLAC: Trial of labor after caesarean section
VBAC: Vaginal births after cesarean section
